# The potential mechanism of Guizhi Fuling Wan effect in the treatment of cervical squamous cell carcinoma: A bioinformatics analysis investigation

**DOI:** 10.1097/MD.0000000000037153

**Published:** 2023-02-02

**Authors:** Xiaoxiang Wang, Tianyue Wang, Xinyu Jiang, Yanmin Ruan, Jiamin Wang, Caixia Qi

**Affiliations:** a The Third Clinical Medical Collage, Zhejiang Chinese Medical University, Hangzhou, China; b The Second Clinical Medical College, Zhejiang Chinese Medical University, Hangzhou, China; c The First Clinical Medical College, Zhejiang Chinese Medical University, Hangzhou, China; d The Fourth Clinical Medical College, Zhejiang Chinese Medical University, Hangzhou, China; e Department of Gynecology and Obstetrics, Zhejiang Provincial People’s Hospital, People’s Hospital of Hangzhou Medical College, Hangzhou, China.

**Keywords:** bioanalysis, cervical squamous cell carcinoma, Guizhi Fuling Wan, molecular docking, network pharmacology, traditional Chinese medicine

## Abstract

As a global malignancy with high mortality rate, targeted drug development for Uterine Cervical Neoplasms is an important direction. The traditional formula Guizhi Fuling Wan (GFW) is widely used in gynecological diseases. However, its potential mechanism of action remains to be discovered. We retrieved GFW and cervical squamous cell carcinoma (CSCC) targets from public databases. The protein–protein interaction network was obtained by string computational analysis and imported Cytoscape_v3.9.0 to obtain the core network and the top 10 Hub genes. Gene Ontology and Kyoto Encyclopedia of Genes and Genomes were used for enrichment analysis of the core network, and then molecular docking to verify whether the selected signaling pathway binds well to the core node. Finally, clinical prognostic analysis and expression differences of Hub genes were validated using the Cancer Genome Atlas database and R language. Our search yielded 152 common targets for GFW and CSCC. The interleukin-17 signaling pathway, tumor necrosis factor signaling pathway, and Toll-like signaling pathway were then selected for further molecular docking from the hub genes enrichment analysis results, which showed good binding. Among the Hub genes, JUN, VEGFA, IL1B, and EGF had a poor prognosis for CSCC. In conclusion, this study illustrates that GFW can have adjuvant therapeutic effects on CSCC through multiple targets and multiple pathways, providing a basis for further research.

## 1. Introduction

Uterine cervical neoplasms, mostly caused by persistent infection of the genital tract with 15 high-risk human papillomavirus types, are the most common malignant neoplasms of the female genital tract.^[1,2]^ According to data provided by the International Agency for Research on Cancer in 2020, cervical cancer is the fourth most common cancer in women, after breast, colon and lung cancer, and is the fourth leading cause of cancerous death in women.^[3]^ Uterine cervical neoplasms are found in the junction of old and new squamous epithelium and columnar epithelium of the uterine cervix, and squamous carcinoma is the most common tumor type, followed by adenocarcinoma and adenosquamous carcinoma.^[4,5]^ Currently, the treatment plan for cervical squamous cell carcinoma (CSCC) is designed mainly based on the tumor stage, with neoadjuvant chemotherapy, radiotherapy and surgical resection complementing each other. Research on targeted drugs has also been the focus of CSCC treatment, and with the development of biological disciplines such as genomics, trials of the combination of multiple targeted drugs tend to transform from single target targets to multi-target networks.^[6]^ In clinical practice, platinum drugs are important adjuvants, and the combination of carboplatin, paclitaxel, and bevacizumab has been shown to be effective.^[7,8]^ This shows that the combination of drugs is a wise disease treatment option and research direction.^[9]^ Chinese medicine has important research value in clinical practice as a coordinated overall multi-pathway and multi-target drug intervention. In recent years, traditional Chinese medicine has gradually been shown to exert significant antitumor and adjuvant effects in the treatment of cervical cancer by inhibiting cancer cell activity, regulating apoptosis, and modulating cell signaling variant pathways.^[10,11]^

Guizhi Fuling Wan (GFW), which is derived from the next volume of Jin Kui Yao by Zhang Zhong Jing, is a herbal formula composed of Cinnamomi Ramulus, Poria Cocos(Schw.) Wolf., Persicae Semen, Paeoniae Radix Alba, and Peony.^[12]^ GFW can resolve blood stasis, regulates Qi and blood, and is nowadays widely used to treat gynecological diseases such as primary dysmenorrhea, polycystic ovary syndrome, and menopausal hot flashes.^[13–15]^ After animal model tests as well as clinical observation and analysis, GFW was found to improve the effects of uterine fibroids.^[16,17]^ Meanwhile, it has been shown that GFW can reduce autophagy in granulosa cells of rats with polycystic ovary syndrome by restoring the PI3K/AKT/mTOR signaling pathway.^[12]^ GFW was also shown to inhibit granulosa cell autophagy in polycystic ovary syndrome via H19/miR-29b-3p.^[18]^ In addition to this, it has now been shown that extracts of GFW might be able to induce inhibition of target protein expression and restore sensitivity in cisplatin-resistant patients.^[19]^ Therefore, GFW has shown its value in oncology treatment. Based on the existing studies of GFW for gynecological diseases and tumors, the present study tried to investigate the potential of GFW for the treatment of CSCC. However, as a compound, GFW has a complex system of chemically active components and potential targets of action, and it is difficult to elaborate its mechanism of disease treatment.^[20]^

**Figure 1.**
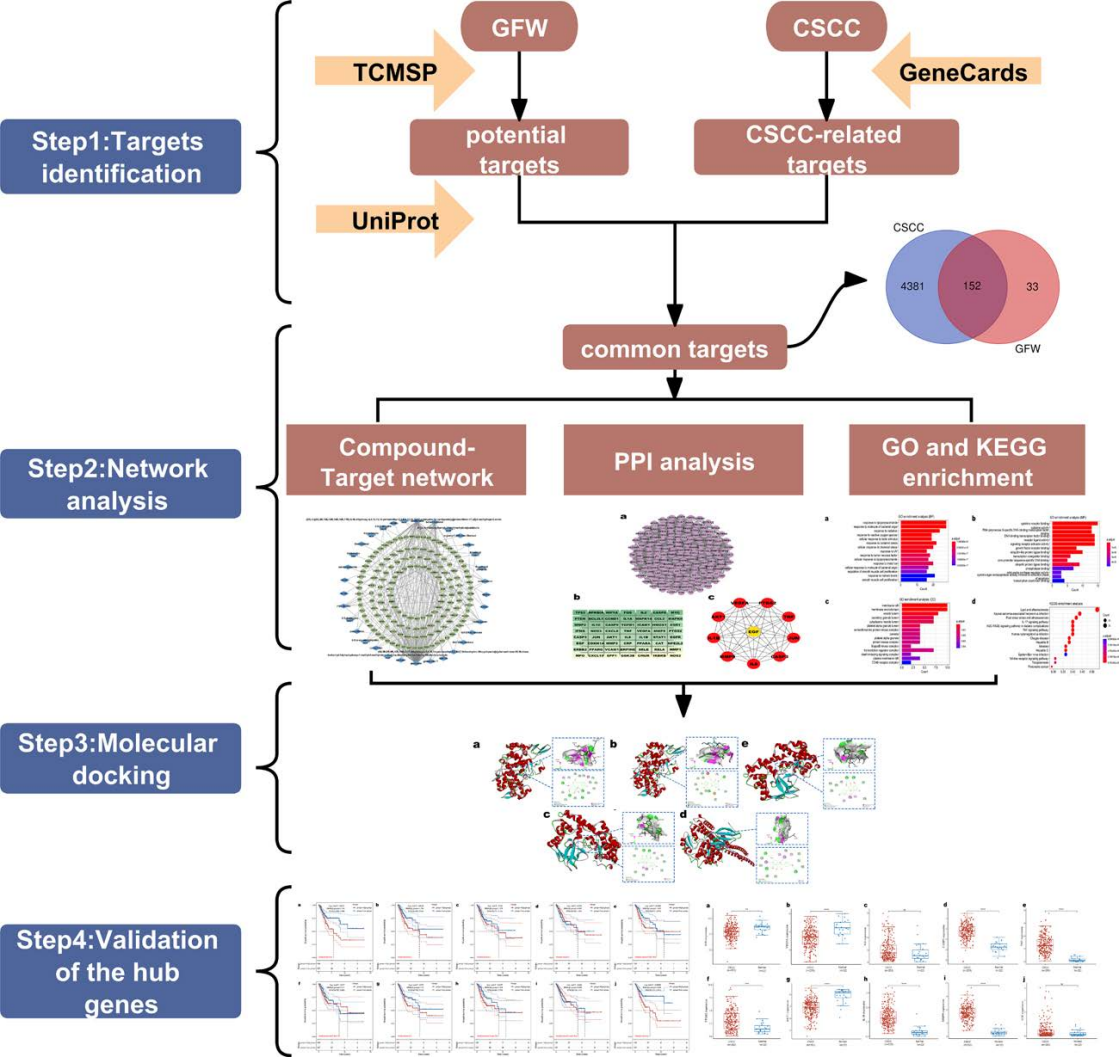
Network pharmacology and bioinformatics workflow diagram.

Network pharmacology is a new field at the intersection of artificial intelligence and medicine, which is based on a large number of medical databases and computer algorithms to construct network relationship diagrams to illustrate the complex mechanisms of drug action.^[21,22]^ The multi-target action in network pharmacology is consistent with the complex mechanism of action of diseases and herbal medicines,^[23]^ which is free from the limitation of “one disease-one target-one drug”^[24]^ and provides new ideas for the study of herbal formulations.^[25]^ In order to extensively verify the pharmacological mechanism of GFW action on CSCC, we will adopt the analytical method of network pharmacology.^[26]^ The detailed workflow diagram of the study is presented in Figure 1.

## 2. Materials and methods

### 2.1. Establish the databases of GFW target genes and CSCC-related genes

The relevant data on herbal medicines and diseases were obtained through the Traditional Chinese Medicine Systems Pharmacology (TCMSP) database (https://old.tcmsp-e.com/tcmsp.php) and the GeneCards database (https://www.genecards.org/).

First, the TCMSP database was applied to search the relevant active ingredients of the 5 Chinese herbal medicines in GFW, and the screening criteria of oral bioavailability ≥30% and drug-likeness ≥0.18 were used to collect the potential action targets of each active ingredient respectively. Then, GeneCards was used to collect CSCC-related genes, and “Cervical Squamous Cell Carcinoma” was used as a keyword to search.

### 2.2. Constructing the relationship between active ingredients of Chinese medicine and diseases

The relevant drug targets collected from TCMSP were imported into the UniProt database (https://www.uniprot.org/) and converted to a common format to align them with the disease targets. GFW and CSCC common genes were obtained by Draw Venn Diagram (https://bioinformatics.psb.ugent.be/webtools/Venn/).

### 2.3. Construction of protein–protein interaction network

Import the GFW and CSCC common genes into the String database (https://cn.string-db.org/), select the “Homo sapiens” species, obtain the protein–protein interaction (PPI) network, and export it in “ tsv “ format file. Afterwards, the files were imported into Cytoscape _ v3.9.0 for visualization and analysis to generate PPI networks.

### 2.4. Acquisition of core networks

The network with the highest score was selected as the core network by analyzing the PPI network with the plug-in MCODE in Cytoscape _ v3.9.0 software. The core network was then analyzed using CytoHubba, a plug-in in Cytoscape _ v3.9.0, to obtain the top 10 ranked Hub genes for follow-up analysis and research.

### 2.5. Gene Ontology and Kyoto Encyclopedia of Genes and Genomes pathway enrichment analysis

The HIPLOT (https://HIPLOT.com.cn) is widely used for bioinformatics analysis as a powerful online data analysis website.^[27]^ We used its Gene Ontology (GO)/Kyoto Encyclopedia of Genes and Genomes (KEGG) enrichment analysis module to investigate the core network. The core genes were imported into the KEGG database in HIPLOT, and the species was selected as “Homo sapiens” with the P threshold set to 0.05. Then the R package “clusterProfiler” was used to enrich the genes to obtain valid information and obtain the results of GO and KEGG analysis.

### 2.6. Molecular docking

We selected from the relevant important signaling pathways enriched by KEGG that are closely related to the disease for the study. The targets of related signaling pathways were collected and collated, and gene targets with links to two or more signaling pathways were selected for follow-up studies. At the same time, the active ingredients represented by the targets were identified according to the previously established database and finally the Chinese herbal medicine to which the active ingredients belonged. The obtained results were imported into Cytoscape _ v3.9.0 software for visual analysis and molecular docking validation.

Molecular docking requires pretreatment of target proteins and small molecules. The protein molecules obtained from the PDB database were modified by PyMOL software. Firstly, we remove the water of crystallization from the protein structure and use the “GetBox Plugin” plug-in to select the active pocket coordinates when the ligand is acquired for docking, then after removing the ligand, we obtain the original ligand of the target protein and export the protein molecule in the pdb. format.

Using AutoDock Tools to add polar hydrogen atoms and charges to proteins and to define atom types. The root of the ligand is determined by the “Torsion Tree” module and a twistable bond suitable for the ligand is selected. After completion, target proteins and small molecules in pdbqt. format will be exported for the next step of molecular docking. The obtained target proteins and small molecules in pdbqt. format will be imported into AutoDock Vina software for molecular docking. To obtain the best conformation of the complex, each molecule will be docked from 10 conformational angles. Finally, Discovery Studio 2020 software was used to perform the constitutive relationship analysis and visualize the results.

### 2.7. Prognostic analysis

We performed a relevant reanalysis and validation of the previously obtained top 10 Hub genes rankings. RNAseq data (level3) and corresponding clinical information of CSCC were obtained from The Cancer Genome Atlas (TCGA) database (https://portal.gdc.cancer.gov/). The log rank was used to test the Kaplan–Meier survival analysis, comparing the survival differences between the above two or more groups, and a timeROC analysis was performed to compare the predictive accuracy of the top 10 ranked Hub genes. For Kaplan–Meier curves, *P* values and hazard ratios with 95% confidence intervals were derived by logrank test and univariate Cox regression. All the above analysis methods and R packages were performed using R software version v4.0.3 (R Foundation for Statistical Computing, 2020). *P* < .05 was considered to be statistically significant.

### 2.8. Validation of differences in expression regarding Hub genes

RNAseq data (level3) and corresponding clinical information for 253 CSCCs were obtained from TCGA dataset (https://portal.gdc.com). We used R software v4.0.3 for statistical analysis and ggplot2 (v3.3.2) to visualize the expression differences of the 10 Hub genes obtained from the analysis. *P* < .05 was considered to be statistically significant.

## 3. Results

### 3.1. Acquisition of GFW active ingredients and gene targets

**Figure 2.**
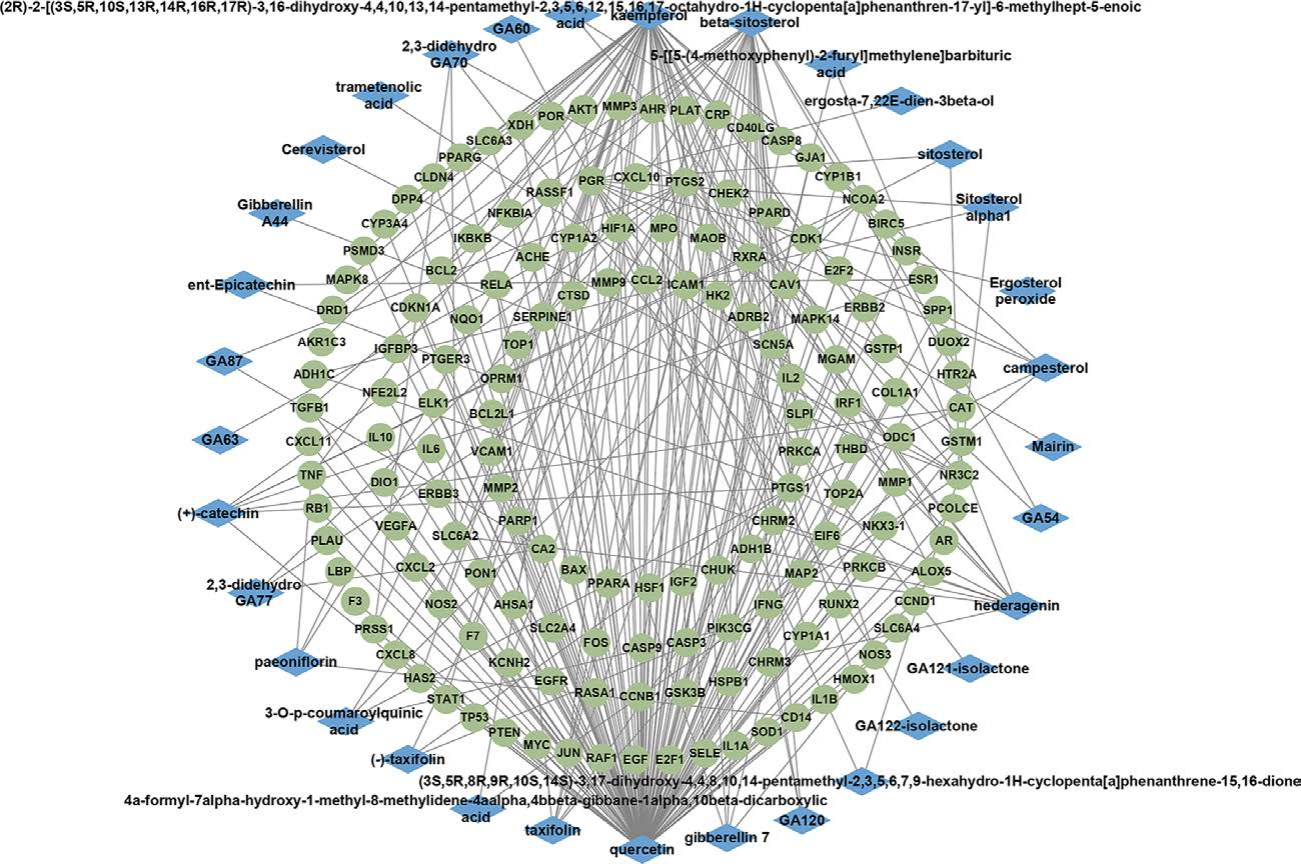
Network diagram of active ingredients of GFW in relation to gene targets. Green circular nodes represent gene targets; blue diamond-shaped nodes represent corresponding active ingredients. GFW = Guizhi Fuling Wan.

The active ingredients of CR, PS, PRA, CM and PC were screened in TCMSP database with oral bioavailability value ≥30% and drug-likeness ≥0.18, respectively. The results showed that there were 58 active ingredients and 185 gene targets in the 5 herbal medicines. The relevant active ingredients are shown in Table 1. The established GFW database was imported into Cytoscape _ v3.9.0 for visualization and analysis, and the active ingredient - gene target relationships were obtained as shown in Figure 2.

**Table 1:** Potentially active compounds in Guizhi Fuling Wan.

No.	Components	OB (%)	DL	Herb
MOL001736	(-)-taxifolin	60.51	0.27	CR
MOL000073	ent-Epicatechin	48.96	0.24	CR
MOL004576	taxifolin	57.84	0.27	CR
MOL011169	Peroxyergosterol	44.39	0.82	CR
MOL000358	beta-sitosterol	36.91	0.75	CR, PS, PRA
MOL000359	sitosterol	36.91	0.75	CR, CM, PRA
MOL000492	(+)-catechin	54.83	0.24	CR, PRA, CM
MOL000273	(2R)-2-[(3S,5R,10S,13R,14R,16R,17R)-3,16-dihydroxy-4,4,10,13,14-pentamethyl-2,3,5,6,12,15,16,17-octahydro-1H-cyclopenta[a]phenanthren-17-yl]-6-methylhept-5-enoic acid	30.93	0.81	PCW
MOL000275	trametenolic acid	38.71	0.8	PCW
MOL000276	7,9(11)-dehydropachymic acid	35.11	0.81	PCW
MOL000279	Cerevisterol	37.96	0.77	PCW
MOL000280	(2R)-2-[(3S,5R,10S,13R,14R,16R,17R)-3,16-dihydroxy-4,4,10,13,14-pentamethyl-2,3,5,6,12,15,16,17-octahydro-1H-cyclopenta[a]phenanthren-17-yl]-5-isopropyl-hex-5-enoic acid	31.07	0.82	PCW
MOL000282	ergosta-7,22E-dien-3beta-ol	43.51	0.72	PCW
MOL000283	Ergosterol peroxide	40.36	0.81	PCW
MOL000285	(2R)-2-[(5R,10S,13R,14R,16R,17R)-16-hydroxy-3-keto-4,4,10,13,14-pentamethyl-1,2,5,6,12,15,16,17-octahydrocyclopenta[a]phenanthren-17-yl]-5-isopropyl-hex-5-enoic acid	38.26	0.82	PCW
MOL000287	3beta-Hydroxy-24-methylene-8-lanostene-21-oic acid	38.7	0.81	PCW
MOL000289	pachymic acid	33.63	0.81	PCW
MOL000290	Poricoic acid A	30.61	0.76	PCW
MOL000291	Poricoic acid B	30.52	0.75	PCW
MOL000292	poricoic acid C	38.15	0.75	PCW
MOL000300	dehydroeburicoic acid	44.17	0.83	PCW
MOL000296	hederagenin	36.91	0.75	PCW,PS
MOL001323	Sitosterol alpha1	43.28	0.78	PS
MOL001328	2,3-didehydro GA70	63.29	0.5	PS
MOL001329	2,3-didehydro GA77	88.08	0.53	PS
MOL001339	GA119	76.36	0.49	PS
MOL001340	GA120	84.85	0.45	PS
MOL001342	GA121-isolactone	72.7	0.54	PS
MOL001343	GA122	64.79	0.5	PS
MOL001344	GA122-isolactone	88.11	0.54	PS
MOL001348	gibberellin 17	94.64	0.49	PS
MOL001349	4a-formyl-7alpha-hydroxy-1-methyl-8-methylidene-4aalpha,4bbeta-gibbane-1alpha,10beta-dicarboxylic acid	88.6	0.46	PS
MOL001350	GA30	61.72	0.54	PS
MOL001351	Gibberellin A44	101.61	0.54	PS
MOL001352	GA54	64.21	0.53	PS
MOL001353	GA60	93.17	0.53	PS
MOL001355	GA63	65.54	0.54	PS
MOL001358	gibberellin 7	73.8	0.5	PS
MOL001360	GA77	87.89	0.53	PS
MOL001361	GA87	68.85	0.57	PS
MOL001368	3-O-p-coumaroylquinic acid	37.63	0.29	PS
MOL001371	Populoside_qt	108.89	0.2	PS
MOL000493	campesterol	37.58	0.71	PS
MOL001910	11alpha,12alpha-epoxy-3beta-23-dihydroxy-30-norolean-20-en-28,12beta-olide	64.77	0.38	PRA
MOL001918	paeoniflorgenone	87.59	0.37	PRA
MOL001919	(3S,5R,8R,9R,10S,14S)-3,17-dihydroxy-4,4,8,10,14-pentamethyl-2,3,5,6,7,9-hexahydro-1H-cyclopenta[a]phenanthrene-15,16-dione	43.56	0.53	PRA
MOL001921	Lactiflorin	49.12	0.8	PRA
MOL001924	paeoniflorin	53.87	0.79	PRA
MOL001928	albiflorin_qt	66.64	0.33	PRA
MOL001925	paeoniflorin_qt	68.18	0.4	PRA, CM
MOL001930	benzoyl paeoniflorin	31.27	0.75	PRA, CM
MOL000211	Mairin	55.38	0.78	PRA, CM
MOL000422	kaempferol	41.88	0.24	PRA, CM
MOL007369	4-O-methylpaeoniflorin_qt	67.24	0.43	CM
MOL007374	5-[[5-(4-methoxyphenyl)-2-furyl]methylene]barbituric acid	43.44	0.3	CM
MOL007382	mudanpioside-h_qt 2	42.36	0.37	CM
MOL007384	paeonidanin_qt	65.31	0.35	CM
MOL000098	quercetin	46.43	0.28	CM

DL = drug-likeness, OB = oral bioavailability.

### 3.2. Common genes of GFW and CSCC

**Figure 3.**
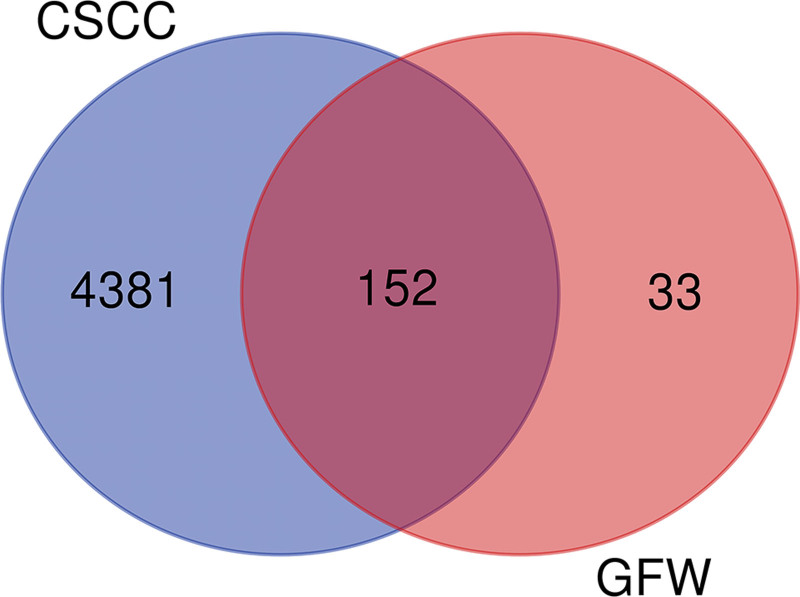
Venn diagram about the common goals of GFW and CSCC. There are 152 common goals between them. CSCC = cervical squamous cell carcinoma, GFW = Guizhi Fuling Wan.

A total of 4533 targets related to CSCC were retrieved using GeneCards. The collected GFW database gene targets were changed into a common format using the UniProt database and then imported into Draw Venn Diagram together with CSCC genes to construct the Venn diagram, and 152 GFW and CSCC common genes were obtained, as shown in Figure 3.

### 3.3. Acquisition of PPI and core networks

**Figure 4.**
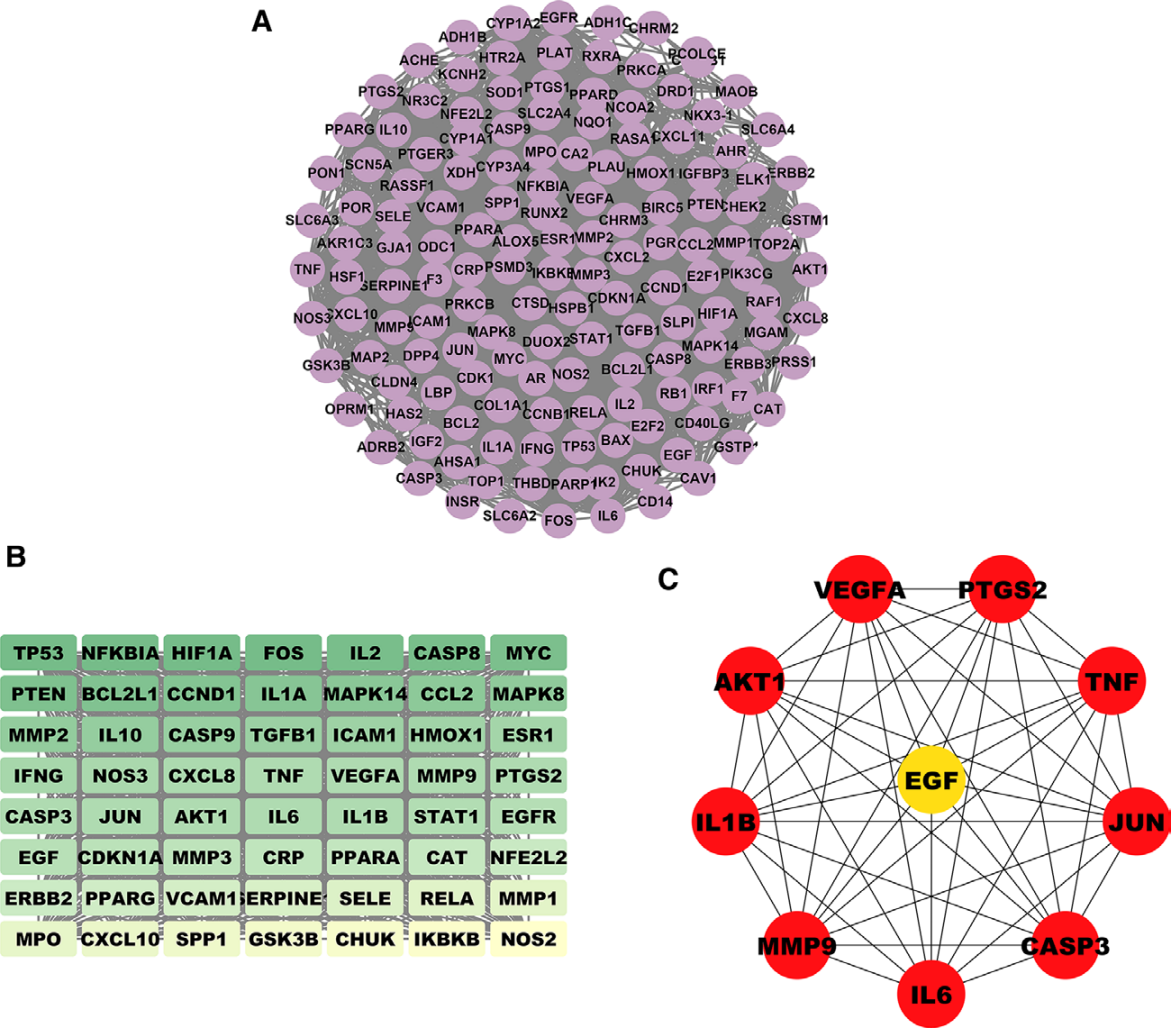
The analysis of the common goal relationship between GFW and CSCC. (A) PPI network diagram. (B) Core network: it consists of a combination of the top 50 common targets, in which the darker the node color, the higher the ranking. (C) The top 10 ranked Hub genes in the core network. Nine red circular nodes represent the highest ranked genes, they are all the top-ranked genes, and the yellow circular nodes in the middle represent the lowest ranked genes. CSCC = cervical squamous cell carcinoma, GFW = Guizhi Fuling Wan, PPI = protein–protein interaction.

As shown in Figure 4A, there are 150 targets and 2770 edges in the PPI network. The PPI network was then further analyzed using the MCODE plug-in, and the network with a score of 45.927 and the highest ranking was selected as the core network, as shown in Figure 4B. This core network has 56 nodes and 1263 edges. The core network was then processed with CytoHubba to obtain the top 10 ranked Hub genes with the highest association in the core network (JUN, VEGFA, IL6, CASP3, TNF, PTGS2, AKT1, IL1B, MMP9, and EGF), which is displayed in Figure 4C.

### 3.4. Results of enrichment analysis of GO and KEGG pathways

**Figure 5.**
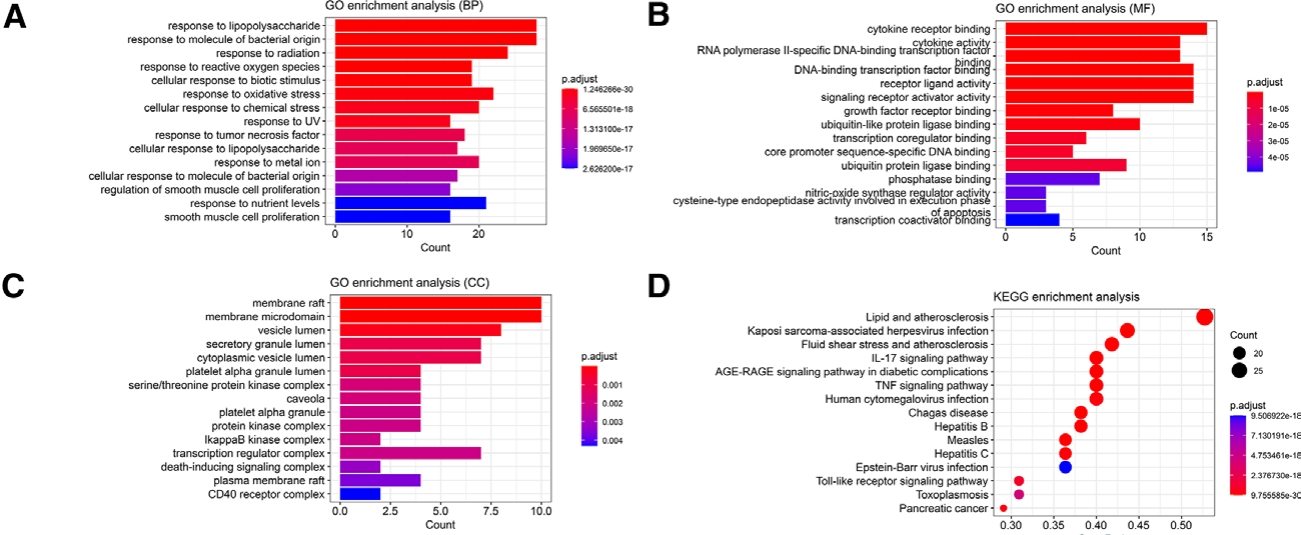
Enrichment analysis of GO and KEGG pathways. (A–C) Results of BP, MF, and CC analyses obtained by GO enrichment, respectively. (D) are the results obtained from KEGG enrichment analysis. In the bar or bubble plots, the smaller the *P*-value, the higher the enrichment c-degree, while the longer the bar or the larger the bubble, the more the number of enriched genes. BP = biological processes, CC = cell composition, GO = gene ontology, KEGG = Kyoto Encyclopedia of Genes and Genomes, MF = molecular functions.

GO and KEGG enrichment analysis of 56 core targets was performed using the HIPLOT online analysis website to explore the mechanistic role of GFW in CSCC treatment. The results of GO analysis are shown in Figure 5A–C, involving biological process, cell composition, and molecular function, respectively. The results of the analysis of the top 15 pathways of KEGG are shown in Figure 5D, where we found that GFW treatment of CSCC was closely associated with IL-17 signaling pathway, tumor necrosis factor (TNF) signaling pathway and Toll-like receptor signaling pathways were closely related.

### 3.5. Molecular docking validation

**Figure 6.**
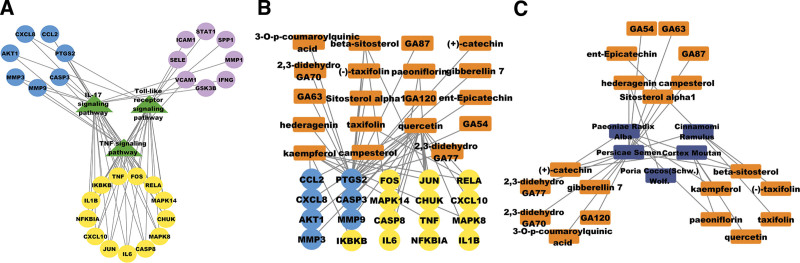
Signaling pathway, target, active ingredients and herbal medicine relationship diagram. (A) Diagram of the relationship between signaling pathways and corresponding targets. Green triangular nodes represent signaling pathways; purple circular nodes represent gene targets that are linked to one of the signaling pathways; blue circular nodes represent gene targets that are linked to two of the signaling pathways; yellow circular nodes represent gene targets that are linked to all 3 signaling pathways. (B) Relationship diagram between gene targets and corresponding active ingredients, orange rectangular nodes represent active ingredients. (C) Relationship diagram between the active ingredient and herbal medicine, dark blue rectangular nodes represent herbal medicine.

Based on the *P*-value and the requirements of the study, we selected IL-17 signaling pathway, TNF signaling pathway and Toll-like receptor signaling pathway for the study. We imported a total of 28 targets from the 3 pathways into Cytoscape _ v3.8.2 for visualization and analysis, and the results are shown in Figure 6.

**Figure 7.**
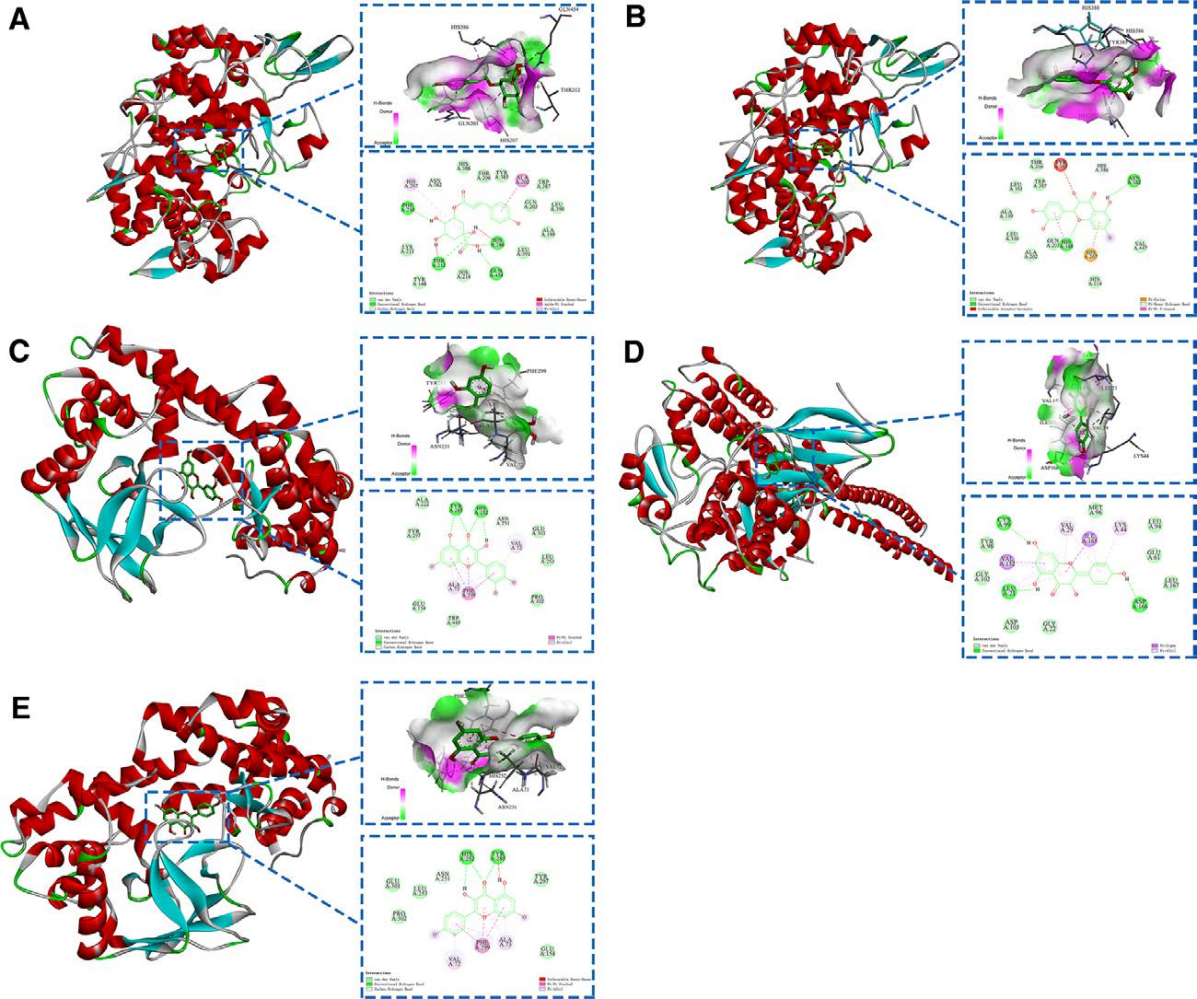
Molecular docking of important targets with corresponding components. (A–E) Top 5 most tightly bound combinations, in the order that 5F19 with 3-O-p-coumaroylquinic acid, 5F19 with (-)-taxifolin, 3QXY with quercetin, 4KIK with kaempferol and 3QXY with kaempferol.

The binding energies of all the targets in the 3 pathways were ranked with the corresponding components, and the results are shown in Table 2. The top 5 combinations ranked from low to high were verified by molecular docking with the software AutoDock Tools, and the visualization of the best conformational fit obtained is presented in Figure 7.

**Table 2:** The combination of the best docking model energy.

Compound	PDB ID	Protein	Affinity (kcal/mol)
PTGS2	5F19	3-O-p-coumaroylquinic acid	−9.4
PTGS2	5F19	(-)-taxifolin	−9.4
RELA	3QXY	quercetin	−9.3
IKBKB	4KIK	kaempferol	−9.3
RELA	3QXY	kaempferol	−9.2
RELA	3QXY	taxifolin	−9
PTGS2	5F19	taxifolin	−9
MMP3	1HY7	quercetin	−9
CHUK	5EBZ	quercetin	−9
PTGS2	5F19	kaempferol	−8.9
PTGS2	5F19	quercetin	−8.9
PTGS2	5F19	ent-Epicatechin	−8.9
PTGS2	5F19	(+)-catechin	−8.7
PTGS2	5F19	Sitosterol alpha1	−8.6
MAPK8	2XRW	kaempferol	−8.6
JUN	2G01	quercetin	−8.6
MAPK14	3DT1	quercetin	−8.5
PTGS2	5F19	campesterol	−8.4
PTGS2	5F19	beta-sitosterol	−8.4
NFKBIA	1IKN	quercetin	−8.4
JUN	2G01	beta-sitosterol	−8.4
FOS	5PAM	quercetin	−8.4
JUN	2G01	kaempferol	−8.3
AKT1	4GV1	quercetin	−8.3
PTGS2	5F19	hederagenin	−8.1
TNF	2AZ5	paeoniflorin	−8
TNF	2AZ5	quercetin	−8
PTGS2	5F19	2,3-didehydro GA77	−7.8
AKT1	4GV1	kaempferol	−7.8
PTGS2	5F19	gibberellin 7	−7.5
CASP3	4PS0	quercetin	−7.4
PTGS2	5F19	GA87	−7.2
PTGS2	5F19	GA63	−7.2
MMP9	4JIJ	quercetin	−7.2
IL1B	5R85	quercetin	−7.2
TNF	2AZ5	kaempferol	−7.1
PTGS2	5F19	GA120	−7.1
PTGS2	5F19	2,3-didehydro GA70	−7
IL6	1N26	quercetin	−7
CASP3	4PS0	beta-sitosterol	−7
PTGS2	5F19	GA54	−6.9
IL6	1N26	paeoniflorin	−6.9
CASP8	4PS1	quercetin	−6.7
CXCL8	6WZM	quercetin	−6.2
CCL2	1DOK	quercetin	−5.9
CXCL10	1O80	quercetin	−5.9

### 3.6. The results of clinical prognostic analysis and expression difference validation regarding Hub genes

**Figure 8.**
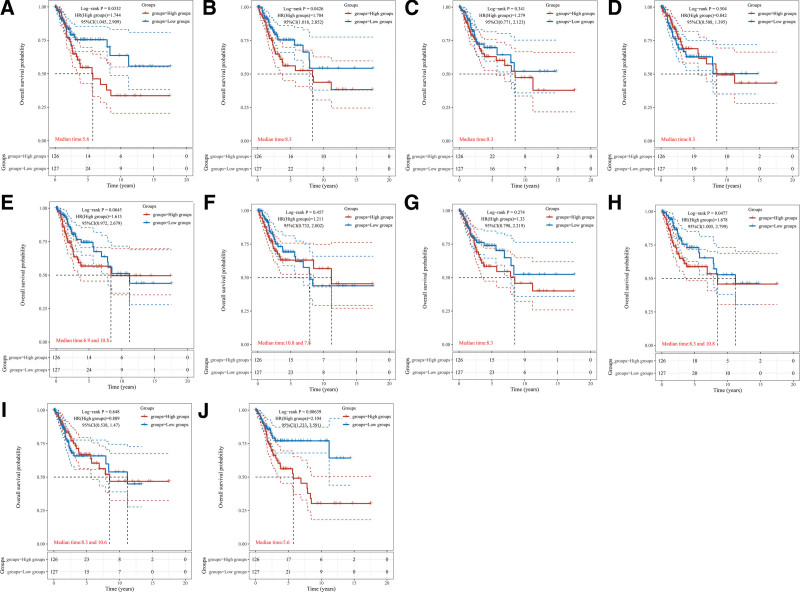
Plot of prognostic analysis of the first 10 Hub genes. The relevant analyses were performed for JUN (A), VEGFA (B), IL6 (C), CASP3 (D), TNF (E), PTGS2 (F), AKT1 (G), IL1B (H), MMP9 (I), and EGF (J), in that order. The HR (High exp) represents the risk coefficient of the high expression group relative to the samples in the low expression group, if HR > 1 means the gene is a risk factor (the higher the expression, the worse the prognosis), if HR < 1 means the gene is a protective factor (the higher the expression, the better the prognosis); 95% CL represents the HR confidence interval; Median time represents the time (i.e., median survival time) corresponding to the survival rate at 50% in both the high expression and low expression groups in years. HR = hazard ratios.

We used the TCGA database to reanalyze the Hub genes and the Kaplan–Meier survival curves obtained are shown in Figure 8. JUN, VEGFA, IL1B, EGF the 4 Hub genes had statistically significant differences in prognostic values, which was *P* < .05. For all 4 genes their hazard ratios was greater than 1, which means that there was a significant negative effect on prognosis.

**Figure 9.**
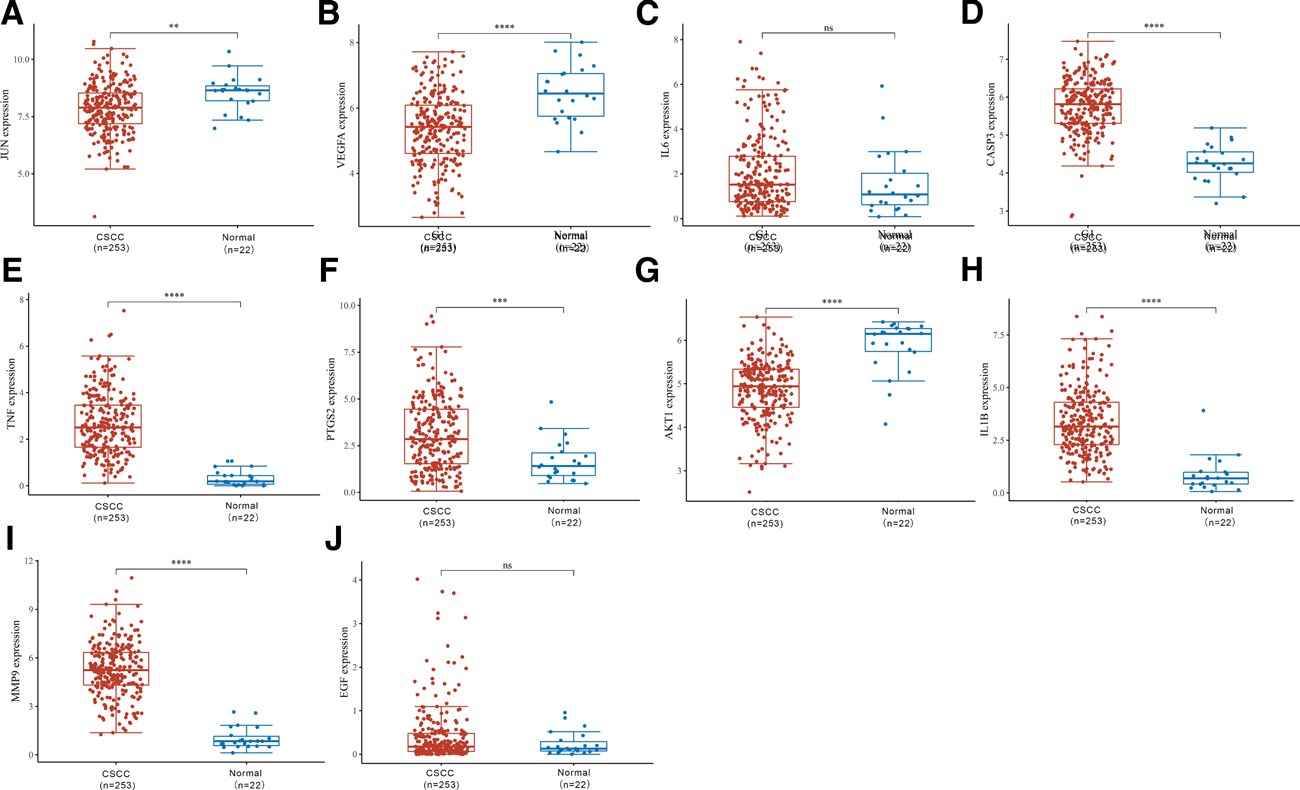
Comparison of gene expression differences. The Wilcox test was used in the significance analysis regarding JUN (A), VEGFA (B), IL6 (C), CASP3 (D), TNF (E), PTGS2 (F), AKT1 (G), IL1B (H), MMP9 (I), and EGF (J). The horizontal coordinates in the graph represent different groups of samples, the vertical coordinates represent the distribution of the expression of this gene, different colors represent different groups, the upper left corner represents the significant *P*-value, *P < .05, **P < .01, ***P < .001, and ****P < .0001.

The differences in gene expression between patients with CSCC and normal subjects are shown in Figure 9. We found significantly lower expression of JUN, VEGFA and AKT1 genes (*P* < .05) and elevated expression of GASP3, TNF, PTGS2, IL1B and MMP9 genes (*P* < .05), while there was no remarkable difference in IL6 and EGF gene expression changes.

## 4. Discussion

First, based on the ADME model, we screened 185 potential GFW targets through the database, and obtained the PPI network with 152 common genes after taking the intersection with the valid targets of CSCC. Then, after obtaining the core network and the top 10 ranked Hub gene, we performed genetic differential analysis and prognostic analysis to verify the correlation between the obtained Hub gene and the disease.

Interestingly, based on the available clinical data, it can be found that the expression of JUN, VEGFA, AKT1, GASP3, TNF, PTGS2, IL1B and MMP9 are significantly different from normal subjects, while the prognosis of JUN, VEGFA, IL1B and EGF is significantly different. For JUN, a proto-oncogene, it is highly susceptible to loss and translocation during malignant tumor development. The expression product of JUN is involved in the composition of transcription factor AP-1, which is associated with important functions such as epithelial cell proliferation, apoptosis and histomorphogenesis.^[28,29]^ The expression of JUN is significantly downregulated in CSCC compared to normal subjects, and the prognosis of the high JUN expression group in CSCC prognostic analysis is poorer, and the expression products of JUN have an influence on the developmental process of CSCC. VEGFA induces tumor angiogenesis by regulating the expression of a series of downstream products to achieve enhanced vascular endothelial cell viability and the formation of an abnormal tumor vascular system. At the same time, it promotes cell migration, inhibits apoptosis and affects tumor growth.^[30–32]^ IL1B, a member of the interleukin family, is a key pro-inflammatory factor involved in cell proliferation, differentiation and apoptosis, which can promote tumor growth and metastasis. High expression of IL1B can lead to poor prognosis in CSCC.^[33,34]^ As for EGFR, it has been an important target for cancer therapy,^[35]^ for which EGF is a ligand. EGF/EGFR has an important role in promoting cell division and enhancing epidermal cell viability. In vitro experiments demonstrated that the growth of Hela cervical cancer cells remained dependent on high EGFR expression.^[36]^ It has been shown that upregulation of EGF expression plays a role in the development of CSCC.^[37]^ The expression of EGF is higher in CSCC than in the general population, and the prognosis of the high expression group is significantly worse. Due to the lack of clinical data, the relevance of some Hub gene to CSCC remains to be explored. The above findings suggest that the potential core targets of GFW action are closely associated with CSCC, and the efficacy of GFW and CSCC has some research value.

The individual targets are interconnected in the core protein network and often act by regulating multiple signaling pathways. Next, we performed KEGG enrichment using core genes. From the top 15 pathways with the highest enrichment scores, IL-17 signaling pathway, TNF signaling pathway, and Toll-like signaling pathway were selected for this study. IL-17 family includes 6 ligand members and 5 receptor members from IL-17A to IL-17F, and the specific proximal adaptor downstream of IL-17R is Act1, which activates downstream pathways including NF-κB, TNF, and Toll through a series of phosphorylation and ubiquitination, activating downstream pathways including NF-κB, MAPKs and C/EBPs^[38–40]^ to regulate cellular activity. Notably, as a classical pro-inflammatory pathway, the IL-17 signaling pathway is also closely associated with tumors, where it can mediate immunosuppression and angiogenesis. A previous study showed that HPV16 oncoprotein can promote angiogenesis and CSCC development by inducing the production of IL-17A+γδ T cells.^[41]^ The TNF signaling pathway cascades with multiple pathways and plays an important role in pathophysiological processes such as cell proliferation, apoptosis, and pro-inflammation.^[42,43]^ Currently, it has been demonstrated that TNF-α can inhibit normal cervical epithelial cells, and stimulate the proliferation of malignant cervical epithelial cells, providing a growth advantage for CSCC.^[44]^ The NF-κB signaling pathway acts as a downstream activation pathway of the TNF signaling pathway,^[45,46]^ where NF-κB has been shown to be constitutively activated during the development of CSCC.^[47]^ The TLR is involved in important molecules in innate immune defense and are thought to have an important role in the clearance of HPV virus infection.^[48]^ In CSCC-related studies, TLR4 upregulation and TLR2, TLR7 downregulation were found to be associated with poor prognosis in CSCC,^[49]^ providing an idea for TLR agonist studies. This can prove that the key signaling pathways enriched in core targets are closely associated with CSCC, and the mechanism of action of GFW has been initially revealed.

Finally, to further validate, we selected 28 core targets of IL-17 signaling pathway, TNF signaling pathway and Toll-like signaling pathway, and molecularly docked them with the corresponding active ingredients of GFW. From the results (Table 2), quercetin, kaempferol, taxifolin and other active ingredients showed high binding efficacy to the relevant targets in the signaling pathway, providing strong evidence for the elucidation of the mechanism of action of GFW on CSCC in terms of molecular docking. Notably, quercetin is an important molecule with anti-tumor effects,^[50]^ and in vitro experiments have demonstrated the potential of quercetin to enhance the sensitivity of human cervical cancer cells to cisplatin-induced apoptosis,^[51]^ playing a potential adjuvant therapeutic role. By establishing a “signaling pathway-active component-Chinese medicine” network, we can find that the 5 major components of GFW, PRA, CR, PS, CM, and PC, all play important roles.

In summary, this study provides a preliminary description of the mechanism of action of GFW on CSCC by establishing a closely linked “active ingredient-target-signaling pathway” network through network pharmacology and bioinformatics analysis. The study demonstrated the potential value of GFW as an adjuvant treatment for CSCC and provided an idea for further research in the future. Of course, this study is based on the existing database and previous studies, and the conclusions obtained lack experimental validation and need to be further explored in the future.

## 5. Conclusion

In this study, we used network pharmacology and molecular docking techniques to verify the specific mechanism of GFW action on CSCC with the concept of “multi-target-multi-pathway.” The results demonstrated that GFW can act on the relevant targets through IL-17 signaling pathway, TNF signaling pathway and Toll-like signaling pathway, and the relevant active ingredients bind well to the important targets in the pathway. This result reveals that GFW has potential adjuvant therapeutic effects on CSCC and provides a theoretical basis for further experimental validation.

## Author contributions

**Conceptualization:** Xiaoxiang Wang, Tianyue Wang, Caixia Qi.

**Funding acquisition:** Caixia Qi.

**Methodology:** Tianyue Wang, Xinyu Jiang, Caixia Qi.

**Software:** Xinyu Jiang, Yanmin Ruan.

**Supervision:** Xiaoxiang Wang.

**Validation:** Xinyu Jiang, Jiamin Wang.

**Visualization:** Xinyu Jiang, Yanmin Ruan.

**Writing – original draft:** Xiaoxiang Wang, Tianyue Wang, Xinyu Jiang, Yanmin Ruan, Caixia Qi.

**Writing – review & editing:** Xiaoxiang Wang, Tianyue Wang, Xinyu Jiang, Yanmin Ruan, Caixia Qi.

## References

[1] Goodman A. HPV testing as a screen for cervical cancer. BMJ. 2015;350:h2372. 26126623 10.1136/bmj.h2372

[2] Marquina G Manzano A Casado A. Targeted agents in cervical cancer: beyond bevacizumab. Curr Oncol Rep. 2018;20:40. 29611060 10.1007/s11912-018-0680-3

[3] Sung H Ferlay J Siegel RL. Global cancer statistics 2020: GLOBOCAN estimates of incidence and mortality worldwide for 36 cancers in 185 countries. CA Cancer J Clin. 2021;71:209–49. 33538338 10.3322/caac.21660

[4] Bhatla N Aoki D Sharma DN. Cancer of the cervix uteri: 2021 update. Int J Gynaecol Obstet. 2021;155(Suppl 1):28–44. 34669203 10.1002/ijgo.13865 PMC9298213

[5] Beckmann MW Stuebs FA Vordermark D.; Collaborators. The diagnosis, treatment, and aftercare of cervical carcinoma. Dtsch Arztebl Int. 2021;118:806–12. 34755595 10.3238/arztebl.m2021.0352 PMC8884069

[6] Gopu P Antony F Cyriac S. Updates on systemic therapy for cervical cancer. Indian J Med Res. 2021;154:293–302. 35295013 10.4103/ijmr.IJMR_4454_20 PMC9131767

[7] Marth C Landoni F Mahner S.; ESMO Guidelines Committee. Cervical cancer: ESMO clinical practice guidelines for diagnosis, treatment and follow-up. Ann Oncol. 2017;28(suppl_4):iv72–83. 28881916 10.1093/annonc/mdx220

[8] Cohen PA Jhingran A Oaknin A. Cervical cancer. Lancet. 2019;393:169–82. 30638582 10.1016/S0140-6736(18)32470-X

[9] Yu L Xia M An Q. A network embedding framework based on integrating multiplex network for drug combination prediction. Brief Bioinform. 2022;23:bbab364. 34505623 10.1093/bib/bbab364

[10] Lin J Chen L Qiu X. Traditional Chinese medicine for human papillomavirus (HPV) infections: a systematic review. Biosci Trends. 2017;11:267–73. 28484110 10.5582/bst.2017.01056

[11] Hsiao YH Lin CW Wang PH. The potential of Chinese herbal medicines in the treatment of cervical cancer. Integr Cancer Ther. 2019;18:1534735419861693. 31271066 10.1177/1534735419861693 PMC6611015

[12] Liu M Zhu H Zhu Y. Guizhi Fuling Wan reduces autophagy of granulosa cell in rats with polycystic ovary syndrome via restoring the PI3K/AKT/mTOR signaling pathway. J Ethnopharmacol. 2021;270:113821. 33460753 10.1016/j.jep.2021.113821

[13] Zhu Y Li Y Liu M. Guizhi Fuling Wan, Chinese herbal medicine, ameliorates insulin sensitivity in PCOS model rats with insulin resistance via remodeling intestinal homeostasis. Front Endocrinol (Lausanne). 2020;11:575. 32973686 10.3389/fendo.2020.00575 PMC7482315

[14] Li M Hung A Li H. A classic herbal formula Guizhi Fuling Wan for menopausal hot flushes: from experimental findings to clinical applications. Biomedicines. 2019;7:60. 31426588 10.3390/biomedicines7030060 PMC6783937

[15] Zhang S Lai X Wang X. Deciphering the pharmacological mechanisms of Guizhi-Fuling capsule on primary dysmenorrhea through network pharmacology. Front Pharmacol. 2021;12:613104. 33746752 10.3389/fphar.2021.613104 PMC7966503

[16] Zhang X Zhao HH Li D. Neuroprotective effects of matrix metalloproteinases in cerebral ischemic rats by promoting activation and migration of astrocytes and microglia. Brain Res Bull. 2019;146:136–42. 30445183 10.1016/j.brainresbull.2018.11.003

[17] Sakamoto S Yoshino H Shirahata Y. Pharmacotherapeutic effects of kuei-chih-fu-ling-wan (keishi-bukuryo-gan) on human uterine myomas. Am J Chin Med. 1992;20:313–7. 1471615 10.1142/S0192415X92000333

[18] Wu P Zhu Y Li J. Guizhi Fuling Wan inhibits autophagy of granulosa cells in polycystic ovary syndrome mice via H19/miR-29b-3p. Gynecol Endocrinol. 2023;39:2210232. 37187204 10.1080/09513590.2023.2210232

[19] Han L Cao X Chen Z. Overcoming cisplatin resistance by targeting the MTDH-PTEN interaction in ovarian cancer with sera derived from rats exposed to Guizhi Fuling wan extract. BMC Complement Med Ther. 2020;20:57. 32066429 10.1186/s12906-020-2825-9 PMC7076886

[20] Wang S Huang J Mao H. A novel method HPLC-DAD analysis of the Contentsof Moutan Cortexand Paeoniae Radix Alba with similar constituents-monoterpene glycosides in Guizhi Fuling Wan. Molecules. 2014;19:17957–67. 25375336 10.3390/molecules191117957 PMC6271269

[21] Wang ZY Wang X Zhang DY. Traditional Chinese medicine network pharmacology: development in new era under guidance of network pharmacology evaluation method guidance. Zhongguo Zhong Yao Za Zhi. 2022;47:7–17. 35178906 10.19540/j.cnki.cjcmm.20210914.702

[22] Jiang X Zhou J Yu Z. Exploration of Fuzheng Yugan mixture on COVID-19 based on network pharmacology and molecular docking. Medicine (Baltimore). 2023;102:e32693. 36701702 10.1097/MD.0000000000032693 PMC9857359

[23] Zhou Z Chen B Chen S. Applications of network pharmacology in traditional Chinese medicine research. Evid Based Complement Alternat Med. 2020;2020:1646905. 32148533 10.1155/2020/1646905 PMC7042531

[24] Nogales C Mamdouh ZM List M. Network pharmacology: curing causal mechanisms instead of treating symptoms. Trends Pharmacol Sci. 2022;43:136–50. 34895945 10.1016/j.tips.2021.11.004

[25] Wang T Zhou Y Wang K. Prediction and validation of potential molecular targets for the combination of Astragalus membranaceus and Angelica sinensis in the treatment of atherosclerosis based on network pharmacology. Medicine (Baltimore). 2022;101:e29762. 35776988 10.1097/MD.0000000000029762 PMC9239660

[26] Wang T Jiang X Ruan Y. Based on network pharmacology and in vitro experiments to prove the effective inhibition of myocardial fibrosis by Buyang Huanwu decoction. Bioengineered. 2022;13:13767–83. 35726821 10.1080/21655979.2022.2084253 PMC9275964

[27] Wang T Jiang X Ruan Y. The mechanism of action of the combination of Astragalus membranaceus and Ligusticum chuanxiong in the treatment of ischemic stroke based on network pharmacology and molecular docking. Medicine (Baltimore). 2022;101:e29593. 35839049 10.1097/MD.0000000000029593 PMC11132396

[28] Tsiambas E Mastronikolis N P Fotiades P. c-Jun/c-Fos complex in laryngeal squamous cell carcinoma. J BUON. 2020;25:618–20. 32521843

[29] Luo A Yu X Li G. Differentiation-associated genes regulated by c-Jun and decreased in the progression of esophageal squamous cell carcinoma. PLoS One. 2014;9:e96610. 24796531 10.1371/journal.pone.0096610 PMC4010476

[30] Claesson-Welsh L Welsh M. VEGFA and tumour angiogenesis. J Intern Med. 2013;273:114–27. 23216836 10.1111/joim.12019

[31] Zhang L Chen Q Hu J. Expression of HIF-2α and VEGF in cervical squamous cell carcinoma and its clinical significance. Biomed Res Int. 2016;2016:5631935. 27413748 10.1155/2016/5631935 PMC4931080

[32] Clere N Bermont L Fauconnet S. The human papillomavirus type 18 E6 oncoprotein induces Vascular Endothelial Growth Factor 121 (VEGF121) transcription from the promoter through a p53-independent mechanism. Exp Cell Res. 2007;313:3239–50. 17678892 10.1016/j.yexcr.2007.06.029

[33] Muhammad SB Hassan F Bhowmik KK. Detection of association of IL1β, IL4R, and IL6 gene polymorphisms with cervical cancer in the Bangladeshi women by tetra-primer ARMS-PCR method. Int Immunopharmacol. 2021;90:107131. 33187912 10.1016/j.intimp.2020.107131

[34] Pontillo A Bricher P Leal VN. Role of inflammasome genetics in susceptibility to HPV infection and cervical cancer development. J Med Virol. 2016;88:1646–51. 26945813 10.1002/jmv.24514

[35] Mehrabi M Mahdiuni H Rasouli H. Comparative experimental/theoretical studies on the EGFR dimerization under the effect of EGF/EGF analogues binding: highlighting the importance of EGF/EGFR interactions at site III interface. Int J Biol Macromol. 2018;115:401–17. 29665393 10.1016/j.ijbiomac.2018.04.066

[36] Hu G Liu W Mendelsohn J. Expression of epidermal growth factor receptor and human papillomavirus E6/E7 proteins in cervical carcinoma cells. J Natl Cancer Inst. 1997;89:1271–6. 9293917 10.1093/jnci/89.17.1271

[37] Okino K Konishi H Doi D. Up-regulation of growth factor receptor-bound protein 10 in cervical squamous cell carcinoma. Oncol Rep. 2005;13:1069–74. 15870923

[38] Amatya N Garg AV Gaffen SL. IL-17 signaling: the Yin and the Yang. Trends Immunol. 2017;38:310–22. 28254169 10.1016/j.it.2017.01.006 PMC5411326

[39] Gaffen SL. Structure and signalling in the IL-17 receptor family. Nat Rev Immunol. 2009;9:556–67. 19575028 10.1038/nri2586 PMC2821718

[40] Gu C Wu L Li X. IL-17 family: cytokines, receptors and signaling. Cytokine. 2013;64:477–85. 24011563 10.1016/j.cyto.2013.07.022 PMC3867811

[41] Van Hede D Polese B Humblet C. Human papillomavirus oncoproteins induce a reorganization of epithelial-associated γδ T cells promoting tumor formation. Proc Natl Acad Sci USA. 2017;114:E9056–65. 29073102 10.1073/pnas.1712883114 PMC5664550

[42] Aggarwal BB Gupta SC Kim JH. Historical perspectives on tumor necrosis factor and its superfamily: 25 years later, a golden journey. Blood. 2012;119:651–65. 22053109 10.1182/blood-2011-04-325225 PMC3265196

[43] Saha P Smith A. TNF-α (tumor necrosis factor-α). Arterioscler Thromb Vasc Biol. 2018;38:2542–3. 30354242 10.1161/ATVBAHA.118.311660

[44] Woodworth CD McMullin E Iglesias M. Interleukin 1 alpha and tumor necrosis factor alpha stimulate autocrine amphiregulin expression and proliferation of human papillomavirus-immortalized and carcinoma-derived cervical epithelial cells. Proc Natl Acad Sci USA. 1995;92:2840–4. 7708734 10.1073/pnas.92.7.2840 PMC42314

[45] Van Quickelberghe E De Sutter D van Loo G. A protein-protein interaction map of the TNF-induced NF-κB signal transduction pathway. Sci Data. 2018;5:180289. 30561431 10.1038/sdata.2018.289 PMC6298254

[46] Pabon NA Zhang Q Cruz JA. A network-centric approach to drugging TNF-induced NF-κB signaling. Nat Commun. 2019;10:860. 30808860 10.1038/s41467-019-08802-0 PMC6391473

[47] Nair A Venkatraman M Maliekal TT. NF-kappaB is constitutively activated in high-grade squamous intraepithelial lesions and squamous cell carcinomas of the human uterine cervix. Oncogene. 2003;22:50–8. 12527907 10.1038/sj.onc.1206043

[48] Halec G Scott ME Farhat S. Toll-like receptors: important immune checkpoints in the regression of cervical intra-epithelial neoplasia 2. Int J Cancer. 2018;143:2884–91. 30121951 10.1002/ijc.31814 PMC6419742

[49] Guleria C Suri V Kapoor R. Human papillomavirus 16 infection alters the Toll-like receptors and downstream signaling cascade: a plausible early event in cervical squamous cell carcinoma development. Gynecol Oncol. 2019;155:151–60. 31375269 10.1016/j.ygyno.2019.07.023

[50] Reyes-Farias M Carrasco-Pozo C. The anti-cancer effect of quercetin: molecular implications in cancer metabolism. Int J Mol Sci. 2019;20:3177. 31261749 10.3390/ijms20133177 PMC6651418

[51] Jakubowicz-Gil J Paduch R Piersiak T. The effect of quercetin on pro-apoptotic activity of cisplatin in HeLa cells. Biochem Pharmacol. 2005;69:1343–50. 15826605 10.1016/j.bcp.2005.01.022

